# Hormonomic Changes Driving the Negative Impact of Broomrape on Plant Host Interactions with Arbuscular Mycorrhizal Fungi

**DOI:** 10.3390/ijms222413677

**Published:** 2021-12-20

**Authors:** Kiril Mishev, Petre I. Dobrev, Jozef Lacek, Roberta Filepová, Bistra Yuperlieva-Mateeva, Anelia Kostadinova, Tsveta Hristeva

**Affiliations:** 1 Institute of Plant Physiology and Genetics, Bulgarian Academy of Sciences, 1113 Sofia, Bulgaria; bistrayuperlieva@yahoo.com (B.Y.-M.); akostadinova_bg@yahoo.co.uk (A.K.); 2 Laboratory of Hormonal Regulations in Plants, Institute of Experimental Botany of the Czech Academy of Sciences, 165 02 Praha, Czech Republic; dobrev@ueb.cas.cz (P.I.D.); lacek@ueb.cas.cz (J.L.); filepova@ueb.cas.cz (R.F.); 3 Tobacco and Tobacco Products Institute, Agricultural Academy, 4108 Plovdiv, Bulgaria

**Keywords:** parasitic plants, mycorrhizal fungi, plant hormones, root exudates, rhizosphere, small-molecule communication, strigolactones

## Abstract

Belowground interactions of plants with other organisms in the rhizosphere rely on extensive small-molecule communication. Chemical signals released from host plant roots ensure the development of beneficial arbuscular mycorrhizal (AM) fungi which in turn modulate host plant growth and stress tolerance. However, parasitic plants have adopted the capacity to sense the same signaling molecules and to trigger their own seed germination in the immediate vicinity of host roots. The contribution of AM fungi and parasitic plants to the regulation of phytohormone levels in host plant roots and root exudates remains largely obscure. Here, we studied the hormonome in the model system comprising tobacco as a host plant, *Phelipanche* spp. as a holoparasitic plant, and the AM fungus *Rhizophagus irregularis*. Co-cultivation of tobacco with broomrape and AM fungi alone or in combination led to characteristic changes in the levels of endogenous and exuded abscisic acid, indole-3-acetic acid, cytokinins, salicylic acid, and orobanchol-type strigolactones. The hormonal content in exudates of broomrape-infested mycorrhizal roots resembled that in exudates of infested non-mycorrhizal roots and differed from that observed in exudates of non-infested mycorrhizal roots. Moreover, we observed a significant reduction in AM colonization of infested tobacco plants, pointing to a dominant role of the holoparasite within the tripartite system.

## 1. Introduction

Rhizosphere is a dynamic platform for complex interactions of plants with the other biotic and abiotic components of the soil ecosystem. The exchange of organic and inorganic matter varies throughout the ontogenesis, and ultimately leads to better adaptation of plants to the fluctuating environment. The chemical composition of root exudates largely shapes the plant-associated microbial communities [1]. The selective enrichment of bacterial species in the rhizosphere is based on their specific substrate preferences secured by the plant species [2]. In general, root exudates contain a variety of primary and secondary metabolites of low molecular weight, as well as macromolecules like proteins and polysaccharides. The exuded chemical components have been shown to be functionally implicated in diverse biological processes such as symbiosis, pathogenesis, allelopathy, and mineral nutrition [3,4]. Importantly, all other biotic factors jointly contribute to the chemical composition in the rhizosphere, and their metabolic activities are subjected to feedback regulation. The rhizosphere microbiome modulates root metabolism and exudation by driving long-distance signaling and systemic transcriptional reprogramming in plants [5].

Beneficial symbiotic interactions between arbuscular mycorrhizal (AM) fungi of the phylum *Glomeromycota* and land plants nicely illustrate the metabolic synchronization and mutual regulation of the processes of growth and stress adaptation. AM fungi gain from host plants photoassimilates and lipids, and in turn supply the plants with mineral nutrients via an extraradical mycelium network [6]. Mineral deficiency, such as phosphate limitation, triggers changes in the root exudate composition that positively influence the AM development for better phosphate acquisition [7]. These precisely orchestrated metabolic readjustments rely on extensive bidirectional small-molecule signaling that underlies all stages of AM symbiosis, including the presymbiotic communication [8].

In the course of land plant evolution, parasitic weeds have abused the chemical communication between host plant roots and beneficial AM fungi to ensure parasitic seed germination. Strigolactones (SLs) are the best studied class of germination stimulants for seeds of holoparasites, such as broomrapes [9,10]. Apart from being phytohormones, SLs are exuded by the host plant to facilitate root colonization with AM fungi via induction of hyphal branching. The core chemical structure of canonical SLs comprises a methyl butenolide ring attached to a hydrophobic tricyclic scaffold via an enol ether bridge. Besides canonical SLs, non-canonical SLs lacking the typical tricyclic scaffold [11], as well as sesquiterpene lactones [12], have also been implicated in broomrape seed germination. Based on their stereochemistry, canonical SLs are subdivided into orobanchol- and strigol-type molecules with species-specific occurrence [9,13,14]. Variations in the levels of the two SL types in the root exudate determine the differential susceptibility of host plants to infestation. For instance, sorghum genotypes with reduced levels of 5-deoxystrigol and increased exudation of orobanchol display *Striga* resistance [15]. In addition, different combinations of SL species in the exudate have unequal efficiency of boosting AM symbiosis and parasitic seed germination, and such uncoupling of the two processes has potential practical applications [16]. In contrast to parasitic plants, SL signaling in autotrophic plants does not seem to be directly related to induction of seed germination. SLs interact with protein receptors with α/β hydrolase activity to initiate a signaling cascade in responsive cells (reviewed in [10,17,18]). In the course of convergent evolution with photosynthetic plants, in holoparasites a subset of karrikin receptors have acquired specificity for recognition of host-derived SL signals [19]. Broadened susceptibility for perception of diverse SL classes is presumably the cause for the recently observed expansion of the plant host preferences of *Orobanche cumana* [20]. Unlike plant systems, the mechanisms of SL sensing by AM fungi are so far unclear.

Plant host interactions with the other biotic factors are mediated by extensive phytohormonal crosstalk. Different hormone signaling pathways share common components to fine-tune the plant response to the changing environment. The latest advances in the studies on hormonal regulation in host plants in the course of root colonization with AM fungi have revealed the complex interplay of growth- and stress-related hormones (reviewed in [21,22,23]). For instance, the host DELLA proteins, negative regulators in the gibberellin (GA) signaling pathway, have been shown to play an essential role for promoting AM development [24]. GAs trigger DELLA polyubiquitination and proteasomal degradation, but other hormones such as abscisic acid (ABA) positively affect DELLA protein functions [25]. Another aspect of the hormonal crosstalk during AM colonization deals with regulation of hormone biosynthesis. The expression of SL biosynthesis-related genes is auxin-dependent, and SL exudation is reduced in conditions of low auxin content, which in turn impairs mycorrhizal colonization [26]. Furthermore, it has been demonstrated that ABA deficiency leads to an increase in the ethylene levels, thus restricting mycorrhization [27]. Hormone partitioning in shoots and roots also appears to be an important determinant for coordinated metabolic interactions between host plants and AM fungi, as has already been shown in experiments with organ-specific cytokinin (CK) depletion [28]. In contrast to mycorrhizal plant systems, much less is known about the influence of obligate parasitic plants on the hormone homeostasis in host plants.

The hormone composition of root exudates is not only a result of the host plant metabolic activity. Recent findings prove that AM fungi produce phytohormones, such as auxins, cytokinins, ethylene, and GA, that might influence both fungal and host plant development [29]. In turn, plant parasites also use hormonal signals for growth regulation. Genetic approaches have elucidated the importance of auxin and ethylene signaling pathways for proper haustorium formation during parasite invasion [30,31]. It is still elusive as to whether parasitic plants exude metabolites with hormonal activity into the rhizosphere.

Here, we used the model AM fungal species *Rhizophagus irregularis* (former name *Glomus intraradices*) and branched broomrape to explore their impact, alone or in combination, on the phytohormone levels in roots and root exudates of a host plant (i.e., oriental tobacco). We found a dominant effect of the parasitic plant on the hormonome in the tripartite system compared to that of the AM fungus. Accordingly, we observed suppressed AM colonization in broomrape-infested tobacco plants. The antagonistic broomrape-tobacco interaction was correlated with diminished levels of exuded SL signals at the tubercle stage of development.

## 2. Results

In our growth assays comprising co-cultivation of tobacco plants with AM spores and/or broomrape seeds, we used perlite as solid substrate, as well as 1/8 strength Murashige and Skoog (MS) medium for irrigation. Such diluted nutrient solution ensured low levels of macronutrients which is essential to enhance the activity of host root exudates for induction of broomrape seed germination and AM hyphal branching [32,33,34]. To better understand the mechanisms of chemical communication within the system host plant – holoparasite – AM fungi, we performed comparative analysis of plant hormone levels in tobacco roots and root exudates. The exudates were collected through incubation of intact roots of individual plants in water for two days (Figure S1). Besides active hormonal species, the applied methodology allowed us to quantify their precursors, catabolites, and conjugated forms [35]. With the exception of ethylene, gibberellins, jasmonates and brassinosteroids, we could detect the presence of the main plant hormone classes in both sample types, i.e., root tissues and exudates. To compare the trends of changes in the different biological repeats irrespective of the natural variation in the absolute amounts of metabolites, the levels of active hormones and their derivatives were calculated relative to those in non-infested and non-inoculated controls. The actual concentrations of all analyzed metabolites are provided in Dataset S1.

### 2.1. Phytohormone Profiling in Infested and Mycorrhizal Host Roots and Exudates

#### 2.1.1. ABA and Related Metabolites

**Figure 1:**
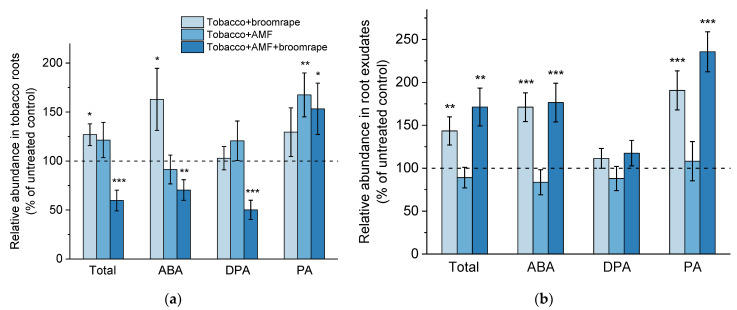
Levels of abscisic acid (ABA) and its catabolites dihydrophaseic acid (DPA) and phaseic acid (PA) in roots (**a**) and exudates (**b**) of tobacco plants upon broomrape infestation and/or colonization with arbuscular mycorrhizal fungi (AMF). The total ABA content represents the sum of all detected ABA-related metabolites in roots and root exudates, respectively (see Section 2.1.1). Values are relative to the non-infested and non-inoculated control (dashed line), and are means of four biological repeats (i.e., independent experiments), with three plants per variant analyzed within each repeat. Error bars indicate standard error of the mean (SE). *** *p* < 0.001, ** *p* < 0.01, and * *p* < 0.05 (Student’s *t*-test).

In our LC-MS analyses, we detected ABA and its catabolites phaseic acid (PA) and dihydrophaseic acid (DPA) in both tobacco root tissues and root exudates, plus 9′-OH ABA in exudate samples. Broomrape infestation of tobacco plants led to a significant increase in the ABA levels in root tissues (Figure 1a). The concentrations of PA, a degradation product with ABA-like biological activity [36], were also elevated in infested roots with or without AM colonization (Figure 1a). PA is generated through ABA oxidation, and is subsequently converted to DPA. Such a rise in the ABA and PA concentrations in infested root samples is in line with recent observations in a similar pathosystem including tomato and *Phelipanche ramosa* [37]. The accumulation of ABA and PA in roots of infested tobacco plants correlated with an increased content of the two metabolites in the growth medium (Figure 1b). Conversely, mycorrhizal roots did not show enhanced accumulation of ABA, and the higher PA amounts in root tissues were not translated into pronounced PA exudation (Figure 1a,b).

#### 2.1.2. Auxins

**Figure 2:**
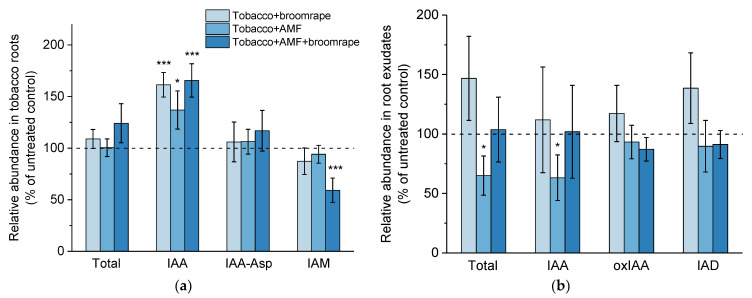
Levels of auxin-related metabolites in roots (**a**) and exudates (**b**) of tobacco plants upon broomrape infestation and/or colonization with arbuscular mycorrhizal fungi (AMF). The total auxin content represents the sum of all detected IAA-related metabolites in roots and root exudates, respectively (see Section 2.1.2). Values are relative to the non-infested and non-inoculated control (dashed line), and are means of four biological repeats (i.e., independent experiments), with three plants per variant analyzed within each repeat. Error bars indicate standard error of the mean (SE). *** *p* < 0.001 and * *p* < 0.05 (Student’s *t*-test). Abbreviations: indole-3-acetic acid (IAA), IAA-aspartate (IAA-Asp), indole-3-acetamide (IAM), oxindole-3-IAA (oxIAA), indole-3-carboxaldehyde (IAD).

Our hormonal profiling of tobacco root samples led to the identification of free indole-3-acetic acid (IAA), the inactive conjugate of IAA with aspartate (IAA-Asp), the catabolite oxindole-3-IAA (oxIAA), as well as the IAA precursor indole-3-acetamide (IAM). Mycorrhizal roots as well as roots co-cultivated with parasitic seeds were characterized by increased content of IAA (Figure 2a). The levels of IAA-Asp and IAM were similar to those in control root samples except for the reduced IAM content measured in mycorrhizal roots with broomrape infestation (Figure 2a).

Furthermore, we detected the presence of three auxin-related metabolites in root exudates: free IAA, oxIAA, and indole-3-carboxaldehyde (IAD), an intermediate in one of the biosynthetic pathways of IAA (reviewed in [38]). It should be noted that the data variation in the concentrations of IAA-related species in all four biological repeats was unusually high. Nevertheless, we observed a clear reduction in the IAA levels in samples with exudates of mycorrhizal plants (Figure 2b). In spite of the higher content of IAA in broomrape-infested roots, their exudates were not significantly enriched in auxin (Figure 2b). There were also no noticeable differences in the amounts of released oxIAA and IAD in all studied samples.

#### 2.1.3. Cytokinins (CKs)

In tobacco root tissues, we detected a number of CK-related metabolites falling into the groups of CK bases (*trans*-zeatin, *cis*-zeatin, dihydrozeatin), CK ribosides (*trans*-zeatin riboside, *cis*-zeatin riboside, dihydrozeatin riboside, isopentenyladenosine), CK *N*-glucosides (*N*7- and *N*9-glucosides of *trans*-zeatin, *cis*-zeatin, dihydrozeatin, and isopentenyladenine), CK *O*-glucosides (*trans*-zeatin-*O*-glucoside, *cis*-zeatin-*O*-glucoside, *cis*-zeatin riboside-*O*-glucoside), and CK phosphates (*trans*-zeatin riboside monophosphate; *cis*-zeatin riboside monophosphate, isopentenyladenosine monophosphate).

**Figure 3:**
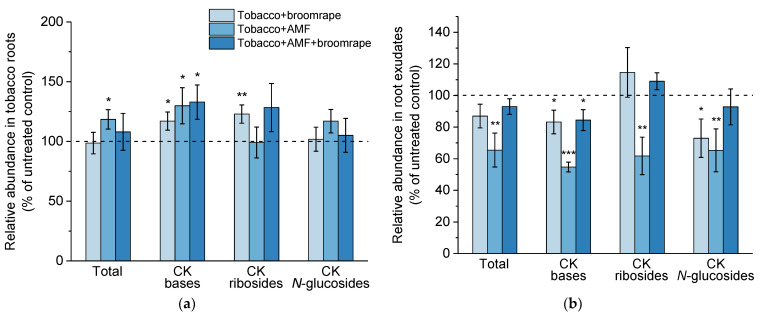
Levels of cytokinin (CK)-related metabolites in roots (**a**) and exudates (**b**) of tobacco plants upon broomrape infestation and/or colonization with arbuscular mycorrhizal fungi (AMF). The total CK content represents the sum of all detected CK-related metabolites in roots and root exudates, respectively (see Section 2.1.3). Values are relative to the non-infested and non-inoculated control (dashed line), and are means of four biological repeats (i.e., independent experiments), with three plants per variant analyzed within each repeat. Error bars indicate standard error of the mean (SE). *** *p* < 0.001, ** *p* < 0.01, and * *p* < 0.05 (Student’s *t*-test).

Among the CK metabolites, the free nucleobases are the only high-affinity ligands for CK receptors in plants [39]. It is now accepted that both *trans*- and *cis*-zeatin forms possess biological activity, the *cis*-isomer role being more pronounced under growth-limiting conditions [40]. CK phosphates are precursors in the CK biosynthetic pathways [41]. CK ribosylation is a reversible modification, and CK nucleosides are the predominant transportation form in plant vascular tissues [41]. In turn, *O*-glucosides are CK storage forms that can also be converted back to active CKs [41]. *N*-glucosides seem to be the prevailing metabolites within the CK pool [42]. Recent findings have revealed that *N*-glucosylation of isopentenyladenine leads to irreversible inactivation, while the *N*-glucosides of *trans*-zeatin can be cleaved back to the active CK nucleobase [43]. As already observed in earlier studies [21,44], the total CK content in mycorrhizal roots was higher compared to that in non-mycorrhizal controls (Figure 3a). In AM colonized roots, we found an increase in the levels of bioactive CK bases, while the amount of CK ribosides and *N*-glucosides remained unaffected. Likewise, the roots of broomrape-infested plants were characterized by higher concentrations of CK bases, but were also enriched in CK nucleosides (Figure 3a).

Regarding the CK profile of root exudates, we detected the presence of CK bases (isopentenyladenine, *cis*-zeatin), CK ribosides (*cis*-zeatin riboside, isopentenyladenosine), CK *N*-glucosides (*N*7-glucosides of *cis*-zeatin and isopentenyladenine), as well as *cis*-zeatin riboside-*O*-glucoside. In contrast to the augmented levels of CK bases detected in mycorrhizal and/or broomrape-infested roots, the corresponding exudates were characterized by diminished presence of these bioactive CK forms (Figure 3b). As a whole, exudates of AM-colonized roots contained considerably less CK-related species, including CK ribosides and *N*-glucosides (Figure 3b).

#### 2.1.4. Phenolic Compounds

**Figure 4:**
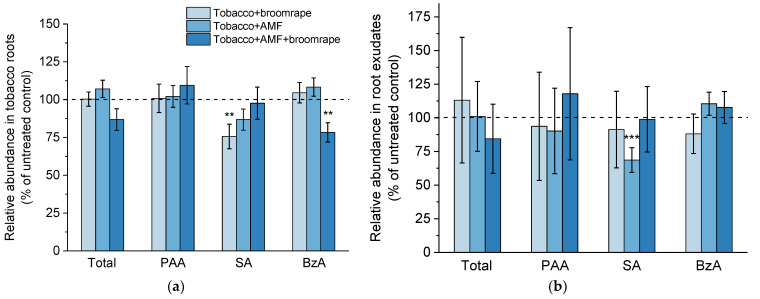
Levels of phenylacetic acid (PAA), salicylic acid (SA), and benzoic acid (BzA) in roots (**a**) and exudates (**b**) of tobacco plants upon broomrape infestation and/or colonization with arbuscular mycorrhizal fungi (AMF). Values are relative to the non-infested and non-inoculated control (dashed line), and are means of four biological repeats (i.e., independent experiments), with three plants per variant analyzed within each repeat. Error bars indicate standard error of the mean (SE). *** *p* < 0.001 and ** *p* < 0.01 (Student’s *t*-test).

Out of the metabolites with phenolic moiety in tobacco root tissues, we analyzed phenylacetic acid (PAA), salicylic acid (SA), and benzoic acid (BzA). The same compounds were found in the medium with exudates where phenylacetamide was also present. PAA has weak auxin activity and, unlike IAA, the PAA concentration gradient along the plant tissues is established by local differences in its biosynthesis rather than polar transport [45]. In turn, BzA is one of the biosynthetic precursors of SA, a key hormone involved in plant defense responses [46,47]. In our hormone profiling assays, we did not observe differential accumulation of PAA in tobacco roots and root exudates as a result of AM colonization and/or broomrape development (Figure 4a,b). Interestingly, the roots of infested plants were characterized by lower SA levels, and co-cultivation with AM spores and broomrape seeds led to reduction in the BzA concentration (Figure 4a). Besides, mycorrhizal roots were found to release significantly less SA into the medium compared to non-inoculated controls (Figure 4b).

### 2.2. Strigolactone (SL) Levels in Root Exudates

SLs are synthesized in extremely low amounts and, in spite of the significant progress in the last few years [48,49], their detection in root tissues is still challenging. Here, we adapted a protocol for purification and quantitative determination of SLs in root exudates of oriental tobacco plants which are known for the very high abundance of secondary metabolites [50] that hamper the efficient detection of poorly represented molecules in the complex exudate matrix. For concentration and purification of the analytes of interest through solid phase extraction (SPE), we first tested the capacity of reversed phase columns with silica-based C18 sorbent to retain SLs. Water solution containing two SL standards, i.e., 2′-*epi*-orobanchol (2′-*epi*-ORB) and 5-deoxystrigol, was applied to preconditioned Sep-Pak^®^ Plus cartridges. Following elution with acetone, aliquots from the loading solution, the flow-through fraction, and the eluate were analyzed by LC-MS/MS (Figure S2). Loss of the methyl butenolide ring upon fragmentation of the protonated molecular ion [M + H]^+^ is associated with the generation of a product ion at *m/z* 97 in the mass spectra which is a characteristic feature of all canonical strigolactones subjected to LC-MS/MS analysis. The absence of detectable SL signals in the flow-through fraction demonstrated efficient retention of the molecules of interest on the column. Peak area quantifications of the amount of 2′-*epi*-ORB and 5-deoxystrigol before SPE and in the final eluate revealed acceptable recovery of 2′-*epi*-ORB (65 %) and 5-deoxystrigol (85 %), albeit lower than recently reported data with polymer-based sorbents with dual retention mode of action [49].

One-step elution of SPE-purified compounds from complex metabolite mixtures, such as root exudates, leads to significant ion suppression during the LC-MS analysis. Hence, stepwise elution with a range of concentrations of the eluting solvent has already been proposed to substantially decrease sample complexity through elimination of highly abundant interfering compounds from the sample matrix [51]. Here, we performed four-step elution of SPE-purified SL standards 2′-*epi*-ORB and GR24 with 25 %, 50 %, 75 %, and 100 % acetone in order to determine the acetone fraction with maximal recovery of the two SL species. The LC-MS analysis of all fractions showed that most of the retained molecules were efficiently eluted with 50 % acetone (Figure S3), a fraction that had previously been shown to have the highest activity in broomrape seed germination and AM hyphal branching assays [52]. Thus, that concentration was chosen for elution of SPE-purified root exudates following a washing step with 25 % acetone for removal of background compounds from the sample.

**Figure 5:**
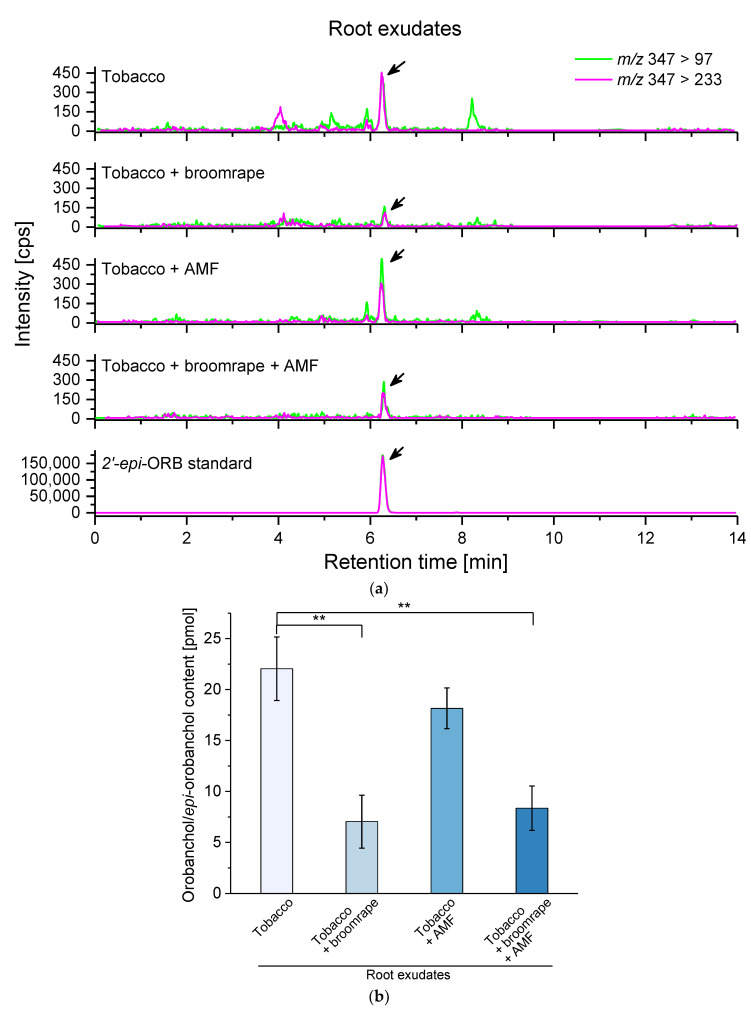
Levels of orobanchol/2′-*epi*-orobanchol (ORB) in exudates of tobacco plants upon broomrape infestation and/or colonization with arbuscular mycorrhizal fungi (AMF). (**a**) Multiple reaction monitoring (MRM) chromatograms showing the ORB-specific transitions of m/z 347 > 97 (green) and 347 > 233 (magenta). The retention time and transitions of 2′-*epi*-orobanchol (2′-*epi*-ORB) standard in water solution are also presented (bottom). (**b**) Quantification of the ORB levels expressed as pmol compound that a plant exudes for 48 h. Values are means of four biological repeats, (i.e., independent experiments), with four plants per variant analyzed within each repeat. Error bars indicate standard error of the mean (SE). ** *p* < 0.01 (Student’s *t*-test) relative to the non-infested and non-inoculated control.

In total, four different batches of plants grown in different seasons were used for collection of root exudates. The presence of tobacco-specific SL species [13] in the exudates was assessed by means of LC-MS/MS in multiple reaction monitoring (MRM) mode. Out of the monitored SLs, we were able to detect clear peaks above the threshold corresponding to orobanchol/2′-*epi*-orobanchol (ORB) based on retention time and characteristic *m/z* transitions 347 > 97 and 347 > 233 (Figure 5a). To take into account the variations due to ion suppression throughout the samples, we added GR24 as internal standard to each of the root exudates prior to SPE. The peak areas of ORB and GR24 in the exudate were normalized to those of the two SLs dissolved in water and processed in the same manner. The quantitative analysis revealed a drastic decrease in the ORB levels measured in root exudates of plants infested with broomrape irrespective of the presence of developing AM fungi in the system (Figure 5b). The amount of ORB in exudates from non-infested mycorrhizal roots was slightly lower than that in non-inoculated controls, the difference being of no statistical significance (Figure 5b).

### 2.3. Impact of Phelipanche on the Germination Stimulant Activity of Root Exudates and AM Development

**Figure 6:**
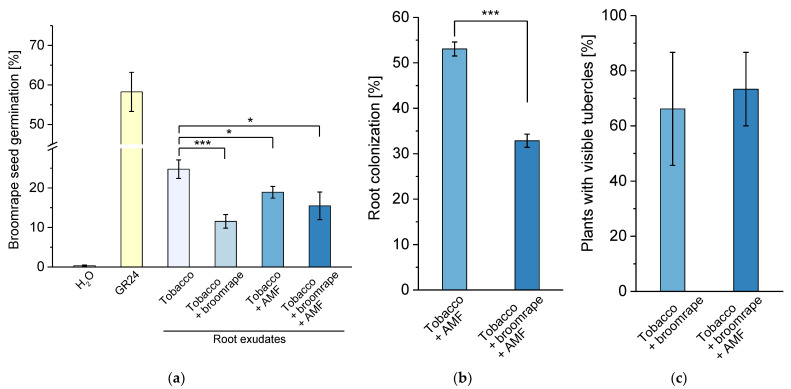
Antagonistic interactions within the system of host plants, parasitic plants, and AM fungi. (**a**) Germination stimulation activity of exudates from infested and/or inoculated tobacco plants on broomrape seeds in *in vitro* conditions. Treatments with water and the synthetic strigolactone GR24 (1.67 µM) were used as negative and positive controls, respectively. (**b**) Extent of mycorrhization of infested vs. non-infested tobacco roots. (**c**) Extent of broomrape infestation of mycorrhizal vs. non-mycorrhizal tobacco roots expressed as percentage of plants with at least one visible tubercle. Values are means of five biological repeats, i.e., independent plant cultivations and exudate collections. Error bars indicate standard error of the mean (SE). *** *p* < 0.001 and * *p* < 0.05 (Student’s *t*-test).

As mentioned above, plant hosts, parasitic plants, and AM fungi jointly contribute to the composition of phytohormones and signaling molecules in the rhizosphere, thus influencing each other’s development. To dissect the impact of every partner in this tripartite model system, we first tested the potential of root exudates from infested and/or mycorrhizal tobacco plants to induce germination of *Phelipanche* seeds in *in vitro* conditions. Treatments with crude exudates from infested plants with and without colonization with *R. irregularis* triggered weaker broomrape seed germination (Figure 6a). This lower potential for stimulation of parasitic seed sprouting is in accordance with the reduced levels of ORB in exudates of infested plants (Figure 5b). The germination stimulant activity of root exudates from mycorrhizal non-infested plants was also lower compared to the non-inoculated controls (Figure 6a) which is in line with earlier observations dealing with similar model systems [53,54].

Next, we analyzed the extent of tobacco root colonization with *R. irregularis* with or without broomrape infestation. We observed a strong reduction in mycorrhization when host plants were co-cultivated with seeds of *Phelipanche* (Figure 6b). Hence, the parasitic plant interferes with AM development. Regarding broomrape development, we could not notice clearly distinguishable changes in the number of host plants with visible tubercles when the root system was inoculated with AM spores (Figure 6c). Nevertheless, an impact of mycorrhizal colonization on tubercle development cannot be ruled out completely as previous studies with a similar pathosystem have revealed a mild decrease in the number of tubercles per plant in case of co-cultivation with AM fungi [53]. Overall, our results revealed the dominant role of *Phelipanche* in the antagonistic interactions within the tripartite system of host plant - parasitic plant - AM fungi.

## 3. Discussion

Since most of the land plants are involved in symbiotic interactions with AM fungi [8], the chemical communication within pathosystems of plant hosts and holoparasites in nature is performed in the context of AM development. Based on carbon costs, host plants regulate the extent of mycorrhization to maintain the balance between beneficial AM symbiosis and AM parasitism [55]. Here, we observed broomrape-induced suppression of AM colonization, suggesting that the holoparasite might disturb the establishment of beneficial plant host-AMF interactions through competition with *R. irregularis* for host-derived photosynthates. Direct physical connections in the tripartite system ensure multidirectional exchange of water and nutrients, but also of small-molecule signals that concomitantly shape the metabolic activity of all partners. SLs are such key chemical signals with a central role for the autoregulation within the system. Their synthesis and exudation by the hosts are finely modulated by other biotic factors and in response to the changing environmental conditions [56,57]. It has already been demonstrated that plants with established AM symbiosis release less SLs in the rhizosphere [54]. Our data revealed a substantial decrease in ORB exudation by broomrape-infested tobacco plants. Like the autoregulation in conditions of excessive AM colonization, the reduced SL release by infested host roots might be an adaptive strategy to suppress further parasitic seed germination. The latter is supported by the lower germination stimulation activity of root exudates from broomrape-infested tobacco plants.

Although the molecular mechanisms underlying the aforementioned feedback inhibition are yet to be explored, the crosstalk with plant defense phytohormones seems to be essential for the adjustment of SL levels. In addition to its prominent role in plant adaptation to abiotic stress conditions, ABA turns out to be intrinsically involved in plant host interactions with AM fungi and holoparasitic plants [21,37,58]. As part of a common module for defense response, JA-, SA-, and ABA-related tomato genes have been shown to be upregulated at the initial stages of interaction of tomato plants with *Phelipanche ramosa* [59]. Interestingly, their upregulation is accompanied by an increase in the expression of SL biosynthetic genes [59]. Moreover, SL-deficient tomato mutants are characterized by reduced levels of JA, SA, and ABA, which renders them more susceptible to fungal pathogens [60]. Like SLs, ABA is synthesized from carotenoid precursors, and the levels of the two signaling molecules are a result of interdependent regulation [61]. It should be noted that the trends of accumulation of endogenous SL hormones and those of exuded SLs might not correlate in the course of broomrape infestation. The latter reflects the dual role of SLs depending on their localization, i.e., growth regulators within plant tissues as well as small-molecule signals with an impact on other organisms in the rhizosphere.

The auxin IAA has also been found to be involved in the regulation of SL exudation. Reduction in the auxin content in an IAA-deficient mutant or after stem girdling leads to a corresponding decrease in the amount of orobanchol and orobanchyl acetate in exudates of pea plants which in turn negatively influences the extent of AM symbiosis [26]. However, plants with non-impaired IAA metabolism and transport show different dynamics of IAA and SL accumulation. In tobacco mycorrhizal roots, we registered higher amounts of IAA, while the ORB levels in the root exudates were slightly lower than those in the non-colonized controls. The same trends were observed in infested versus non-infested roots and root exudates. An increase in auxin content in mycorrhizal roots has been documented in several plant species except for tobacco [62,63]. The absence of an effect of *R. irregularis* on tobacco root auxin content reported in [63] might be due to age-dependent specificities in host plant response to AM colonization, as the hormonal analyses have been done with young tobacco plants at an early stage of AM symbiosis.

The AM-induced accumulation of auxin in roots appears to induce local responses essential for AM development. Such cell type-specific reprogramming triggered by auxin has been demonstrated for arbuscule-containing host root cortical cells [62]. Likewise, auxin-mediated reprogramming possibly takes place at the site of broomrape attachment to the host root in the course of infestation. Cytokinins are another class of plant hormones that act in concert with auxins to shape plant development in optimal and suboptimal conditions [64]. Analogously to IAA, we detected an increase in the content of bioactive CK bases in root tissues of mycorrhizal plants as well as in broomrape-infested samples. The rise in CK levels might confer limitation of excessive mycorrhization and might also restrict the further spread of broomrape infestation. Such a hypothesis is supported by previous findings demonstrating that CKs play a central role in coordinating the extent of AM colonization with the shoot and root growth of host plants through regulation of the exchange of carbon and phosphate between the two partners [28].

Interestingly, the increased abundance of IAA and CK bases in host roots co-cultivated with *Phelipanche* and/or *R. irregularis* did not result in respective enrichment of those hormonal species in the root exudates. Moreover, the levels of IAA and all studied CKs were consistently lower in exudates of AM colonized roots, a trend that was partially reverted when broomrape was also present in the system. The functional significance of exuded auxins and CKs has yet to be elucidated. Recent studies have demonstrated that host-derived CKs can be released into the growth medium to serve as molecular signals for induction of haustorium formation of *P. ramosa* [65]. Besides, *R. irregularis* also contributes to the CK composition of the growth medium as germinated spores have been shown to release at least one CK metabolite (i.e., isopentenyladenosine) [29]. In turn, all three partners in the system host-parasite-AM fungus produce auxin and possibly contribute to the auxin levels measured in the medium [29,31]. In *Phelipanche*, the processes of hormone production and exudation are poorly explored. Whole-genome sequencing of representatives of the *Orobanchaceae* family should shed light on which of the conventional pathways for phytohormone biosynthesis are functional in broomrapes.

**Figure 7:**
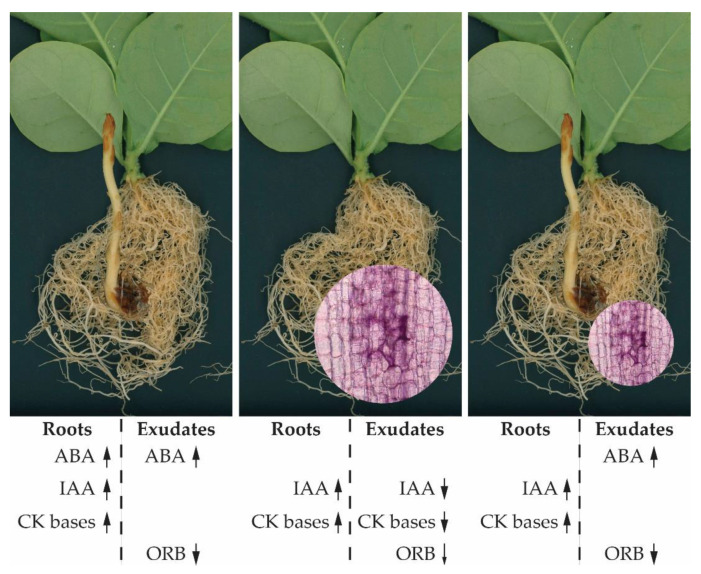
Overview of the host plant-parasitic plant-AM fungi interactions associated with plant hormone regulation. Shown are characteristic trends in the levels of endogenous and exuded hormonal species found in this study. Upwards and downwards arrows depict an increase or decrease in the hormonal content, respectively (the thinner arrow next to ORB in exudates of mycorrhizal plants represents mild reduction without statistical significance). Note the weaker root colonization with *R. irregularis* in broomrape-infested tobacco plants.

In conclusion, we managed to identify previously undescribed hormone-related metabolites present in tobacco root exudates. The comparative analysis in roots and root exudates revealed characteristic hormonal profiles associated with the impact of mycorrhization as well as with broomrape development. For most of the studied metabolites, the trends detected upon co-cultivation of tobacco concomitantly with AM spores and broomrape seeds resembled those registered in samples from broomrape-infested non-inoculated plants, an observation pointing to a dominant effect of broomrape over the AM fungus within the tripartite system. A schematic summary of the most characteristic changes in the plant hormonome identified in this study is provided in Figure 7. In the long term, these findings might expand the possibilities for modulating the communication in the host plant-parasitic plant-AM fungi system in search for advanced strategies for improvement of host plant tolerance to *Phelipanche* infestation.

## 4. Materials and Methods

### 4.1. Plant and Fungal Material

Seeds of oriental tobacco (*Nicotiana tabacum* L., cultivar Krumovgrad 90) were sourced from the collection of the Tobacco and Tobacco Products Institute. Broomrape seeds were harvested from infested tobacco fields in the region of Plovdiv, Bulgaria (42°04′55.2” N, 24°42′16.8” E), and were a mixed population of *Phelipanche ramosa* (L.) Pomel and *Phelipanche mutelii* (Schultz) Pomel [66]. Spores of the AM fungus *Rhizophagus irregularis* (also known as *Glomus intraradices*) were purchased as ready-to-use MycoPlant^®^ inoculant from Tratamientos Bio-Ecológicos, S.A (San Javier, Spain).

### 4.2. Growth Conditions and Preparation of Root Exudates

Tobacco seeds were sown on water-soaked perlite and germinated for three weeks in a growth chamber at 22°C and 60% relative humidity under a 16-h light/8-h dark cycle (white light emitted by fluorescent lamps with intensity of 130 µmol m^−2^ s^−1^) followed by transfer to individual pots with diameter 5.5 cm filled with perlite that was prewetted with 1/8 strength Murashige and Skoog (MS) medium (pH 5.7). At that stage, seeds of *Phelipanche* spp. and/or spores of *R. irregularis* were added to the growth substrate proximal to the roots of the transplanted seedlings. The plants were further grown under the aforementioned conditions for 40 to 50 days and watered weekly with 1/8 MS medium. In total, 35 plants per treatment were cultivated within each biological repeat to ensure enough material for all metabolomic and phenotyping assays described below. For collection of root exudates from individual plants, the perlite was removed and the intact plants with rinsed roots were transferred to glass beakers where the root system was covered with 100 mL of distilled water. After 48-hour incubation in the same growth chamber, the water solution was passed through paper filters (2 µm pore size) and further processed for purification of plant hormones.

### 4.3. Determination of Plant Hormones in Root Exudates

The endogenous phytohormones in crude root exudates were determined according to Prerostova et al. [67]. In brief, 50 uL aliquots of root exudates were spiked with stable isotope-labeled internal standards (1 pmol/sample) and directly used for LC/MS. The phytohormonal metabolites were separated on Kinetex EVO C_18_ column (2.6 µm, 150 × 2.1 mm, Phenomenex, Torrance, CA, USA). The mobile phase A contained 5 mM ammonium acetate and 2 µM medronic acid in water, while phase B consisted of 95 % (*v/v*) acetonitrile in water. The following gradient was used: 5% B in 0 min, 5–7% B (0.1–5 min), 10–35% B (5.1–12 min) and 35–100% B (12–13 min), followed by a 1 min hold at 100% B (13–14 min) and return to 5% B. Hormone analysis was done with an LC/MS system consisting of UHPLC 1290 Infinity II (Agilent, Santa Clara, CA, USA) coupled to 6495 Triple Quadrupole Mass Spectrometer (Agilent, Santa Clara, CA, USA), operating in multiple reaction monitoring (MRM) mode, with quantification by the isotope dilution method. Data acquisition and processing was performed with Mass Hunter software B.08 (Agilent, Santa Clara, CA, USA).

### 4.4. Strigolactone Purification and Quantification in Root Exudates

SL content determination was done in freshly collected root exudates from individual plants. To each sample, racemic GR24 (Chiralix, Nijmegen, Netherlands) was added as internal reference at a final concentration of 100 nM. The exudates were then subjected to solid phase extraction (SPE) using Sep-Pak Plus Short tC18 columns with 400 mg sorbent (Waters). To reduce the matrix effect, stepwise elution with 25 % and 50 % acetone was undertaken, and the 50 % acetone fraction was further used for SL analysis. A standard water solution containing GR24 (100 nM) and 2′-*epi*-orobanchol (500 nM, OlChemIm s.r.o., Olomouc, Czech Republic) was processed in the same way as the root exudates. The acetone was evaporated, and the dry matter in the samples was re-dissolved in 50 % acetonitrile in water prior to loading for LC-MS/MS. The chromatographic separation was performed on Kinetex C18 column (2.6 µm, 100 × 3 mm, Phenomenex) at a rate of 0.3 mL/min. Mobile phases consisted of A: 50 mM acetic acid in water, B: water, and C: acetonitrile:water (95:5, *v/v*). Mobile phase A at 5% was kept constant throughout the run. The gradient program was as follows: 25–95% C in 7 min, followed by 2 min hold at 95% C, and return to 25% C for 1 min with a total cycle time of 14 min. The MS was set up in positive electrospray ionization mode. SL content was analyzed in MRM mode. The retention times and MRM transitions from root exudates were compared to those of synthetic SL standards (OlChemIm): 2′-*epi*-orobanchol (diagnostic MRM *m/z* transitions 347 > 97 and 347 > 233), orobanchyl acetate (389 > 97, 389 > 233), and 5-deoxystrigol (331 > 97 and 331 > 217). The most intense MRM transition was used for quantitative assessment of SL levels.

### 4.5. Plant Hormone Profiling in Tobacco Roots

Within each biological repeat, part of the plants not used for root exudate collection were cleaned from perlite, and the distal end of the root system was sectioned, weighed, and immediately frozen in liquid nitrogen (approximately 150 mg fresh weight/sample). The root material was homogenized with zirconium oxide beads (5 mm diameter) in TissueLyser LT (Qiagen) for 2 min at 50 Hz. Plant hormone extraction and purification was performed as described before [68,69]. In brief, cold extraction solvent consisting of methanol/water/formic acid in a 15:4:1 (*v/v/v*) ratio was added to the homogenized root material together with a mixture of stable isotope-labeled internal standards (10 pmol/sample). SPE was performed using Oasis MCX columns (Waters) with mixed-mode polymeric sorbent (30 mg) and yielded two fractions: the first one eluted with methanol containing metabolites of acidic and neutral character (incl. auxins, ABA, SA, JA), and the second one eluted with 0.35 M ammonium hydroxide in 70 % methanol containing basic compounds (incl. CKs). Following evaporation, the first fraction was redissolved in 15 % acetonitrile in water, while the basic fraction was redissolved in 5 % methanol in water. Fractions were analyzed by HPLC (Ultimate 3000, Dionex, Sunnyvale, CA, USA) coupled to the 3200 Q TRAP hybrid triple quadrupole/linear ion trap mass spectrometer (Applied Biosystems, Waltham, MA, USA). The LC-MS conditions were set as described previously [69]. The hormones were quantified by the isotope dilution method with multilevel calibration curves (*r*² > 0.99). Data processing was carried out with Analyst 1.5 software (Applied Biosystems).

### 4.6. Phelipanche Seed Germination Tests

Broomrape seeds were surface-sterilized by incubation for 10 min in a solution of absolute ethanol and sodium hypochlorite (12 % Cl) mixed in a 4:1 (*v/v*) ratio followed by 3 rinses with absolute ethanol. The air-dried sterilized seeds were sown on a Whatman GF/C filter (1 x1 cm size) soaked with autoclaved water and then covered with another GF/C filter of the same size. Six to seven of the seed-containing stacks were placed in a 9 cm round Petri dish, sealed with parafilm and incubated for seven days at 24 °C in the dark for seed conditioning. At the end of the seed imbibition period, the stacks were transferred to a new Petri dish, the water was left to evaporate for 15 to 20 min in a clean bench, and replaced by a solution of root exudates (200 µL solution per stack). The Petri dish was again sealed with parafilm and incubated for another 7 days at 24 °C in the dark. The seeds were then separated from the filter paper and distributed in small portions in water drops to facilitate the subsequent counting of the percentage of germinated seeds out of the total number of sown seeds. Treatment with distilled water was done as negative control. Positive control experiments were done with racemic GR24 prepared as 1.67 µM solution in 0.05 % acetone. The germination of at least 200 broomrape seeds per treatment was inspected within each of the five biological repeats.

### 4.7. Mycorrhization Analysis

After perlite removal, the roots were cleared and stained with 0.05 % trypan blue as described previously [70]. Tobacco root colonization by *R. irregularis* was quantified by means of the gridline intersect method [71]. The roots of 10 plants were analyzed within each of the five biological repeats.

### 4.8. Statistical Analysis

*P* values were calculated with a two-tailed Student’s *t*-test using Excel software. The sample size and number of independent biological repeats for each type of analysis are provided above.

## Data Availability

The data described in this study can be found in the article and the Supplementary Material. The seed materials are available upon request from the corresponding authors Kiril Mishev and Tsveta Hristeva.

## Supplementary Materials

The following are available online at https://www.mdpi.com/article/10.3390/ijms222413677/s1.

## References

[1] Hu L.F., Robert C.A.M., Cadot S., Zhang X., Ye M., Li B.B., Manzo D., Chervet N., Steinger T., van der Heijden M.G.A. (2018). Root exudate metabolites drive plant-soil feedbacks on growth and defense by shaping the rhizosphere microbiota. Nat. Commun..

[2] Zhalnina K., Louie K.B., Hao Z., Mansoori N., da Rocha U.N., Shi S.J., Cho H.J., Karaoz U., Loque D., Bowen B.P. (2018). Dynamic root exudate chemistry and microbial substrate preferences drive patterns in rhizosphere microbial community assembly. Nat. Microbiol..

[3] Haichar F.E., Santaella C., Heulin T., Achouak W. (2014). Root exudates mediated interactions belowground. Soil Biol. Biochem..

[4] van Dam N.M., Bouwmeester H.J. (2016). Metabolomics in the rhizosphere: Tapping into belowground chemical communication. Trends Plant Sci..

[5] Korenblum E., Dong Y.H., Szymanski J., Panda S., Jozwiak A., Massalha H., Meir S., Rogachev I., Aharoni A. (2020). Rhizosphere microbiome mediates systemic root metabolite exudation by root-to-root signaling. Proc. Natl. Acad. Sci. USA.

[6] Giovannini L., Palla M., Agnolucci M., Avio L., Sbrana C., Turrini A., Giovannetti M. (2020). Arbuscular mycorrhizal fungi and associated microbiota as plant biostimulants: Research strategies for the selection of the best performing inocula. Agronomy.

[7] Bouwmeester H.J., Roux C., Lopez-Raez J.A., Becard G. (2007). Rhizosphere communication of plants, parasitic plants and AM fungi. Trends Plant Sci..

[8] Choi J., Summers W., Paszkowski U. (2018). Mechanisms underlying establishment of arbuscular mycorrhizal symbioses. Annu. Rev. Phytopathol..

[9] Bouwmeester H., Li C.S., Thiombiano B., Rahimi M., Dong L.M. (2021). Adaptation of the parasitic plant lifecycle: Germination is controlled by essential host signaling molecules. Plant Physiol..

[10] Nelson D.C. (2021). The mechanism of host-induced germination in root parasitic plants. Plant Physiol..

[11] Ueno K., Furumoto T., Umeda S., Mizutani M., Takikawa H., Batchvarova R., Sugimoto Y. (2014). Heliolactone, a non-sesquiterpene lactone germination stimulant for root parasitic weeds from sunflower. Phytochemistry.

[12] Raupp F.M., Spring O. (2013). New sesquiterpene lactones from sunflower root exudate as germination stimulants for *Orobanche cumana*. J. Agric. Food Chem..

[13] Xie X.N., Yoneyama K., Kisugi T., Uchida K., Ito S., Akiyama K., Hayashi H., Yokota T., Nomura T., Yoneyama K. (2013). Confirming stereochemical structures of strigolactones produced by rice and tobacco. Mol. Plant.

[14] Brun G., Braem L., Thoiron S., Gevaert K., Goormachtig S., Delavault P. (2018). Seed germination in parasitic plants: What insights can we expect from strigolactone research?. J. Exp. Bot..

[15] Gobena D., Shimels M., Rich P.J., Ruyter-Spira C., Bouwmeester H., Kanuganti S., Mengiste T., Ejeta G. (2017). Mutation in sorghum *LOW GERMINATION STIMULANT 1* alters strigolactones and causes *Striga* resistance. Proc. Natl. Acad. Sci. USA.

[16] Cardoso C., Charnikhova T., Jamil M., Delaux P.M., Verstappen F., Amini M., Lauressergues D., Ruyter-Spira C., Bouwmeester H. (2014). Differential activity of *Striga hermonthica* seed germination stimulants and *Gigaspora rosea* hyphal branching factors in rice and their contribution to underground communication. PLoS ONE.

[17] Burger M., Chory J. (2020). The many models of strigolactone signaling. Trends Plant Sci..

[18] Machin D.C., Hamon-Josse M., Bennett T. (2020). Fellowship of the rings: A saga of strigolactones and other small signals. New Phytol..

[19] Conn C.E., Bythell-Douglas R., Neumann D., Yoshida S., Whittington B., Westwood J.H., Shirasu K., Bond C.S., Dyer K.A., Nelson D.C. (2015). Convergent evolution of strigolactone perception enabled host detection in parasitic plants. Science.

[20] Dor E., Plakhine D., Joel D.M., Larose H., Westwood J.H., Smirnov E., Ziadna H., Hershenhorn J. (2020). A new race of sunflower broomrape (*Orobanche cumana*) with a wider host range due to changes in seed response to strigolactones. Weed Sci..

[21] Liao D.H., Wang S.S., Cui M.M., Liu J.H., Chen A.Q., Xu G.H. (2018). Phytohormones regulate the development of arbuscular mycorrhizal symbiosis. Int. J. Mol. Sci..

[22] Gutjahr C. (2014). Phytohormone signaling in arbuscular mycorhiza development. Curr. Opin. Plant Biol..

[23] Ludwig-Müller J. (2020). Auxins and other phytohormones as signals in arbuscular mycorrhiza formation. The Model Legume Medicago Truncatula.

[24] Floss D.S., Levy J.G., Levesque-Tremblay V., Pumplin N., Harrison M.J. (2013). DELLA proteins regulate arbuscule formation in arbuscular mycorrhizal symbiosis. Proc. Natl. Acad. Sci. USA.

[25] Achard P., Cheng H., de Grauwe L., Decat J., Schoutteten H., Moritz T., van der Straeten D., Peng J.R., Harberd N.P. (2006). Integration of plant responses to environmentally activated phytohormonal signals. Science.

[26] Foo E. (2013). Auxin influences strigolactones in pea mycorrhizal symbiosis. J. Plant Physiol..

[27] Martin-Rodriguez J.A., Leon-Morcillo R., Vierheilig H., Ocampo J.A., Ludwig-Muller J., Garcia-Garrido J.M. (2011). Ethylene-dependent/ethylene-independent ABA regulation of tomato plants colonized by arbuscular mycorrhiza fungi. New Phytol..

[28] Cosme M., Ramireddy E., Franken P., Schmulling T., Wurst S. (2016). Shoot- and root-borne cytokinin influences arbuscular mycorrhizal symbiosis. Mycorrhiza.

[29] Pons S., Fournier S., Chervin C., Becard G., Rochange S., Frey N.F.D., Pages V.P. (2020). Phytohormone production by the arbuscular mycorrhizal fungus *Rhizophagus irregularis*. PLoS ONE.

[30] Cui S.K., Kubota T., Nishiyama T., Ishida J.K., Shigenobu S., Shibata T.F., Toyoda A., Hasebe M., Shirasu K., Yoshida S. (2020). Ethylene signaling mediates host invasion by parasitic plants. Sci. Adv..

[31] Ishida J.K., Wakatake T., Yoshida S., Takebayashi Y., Kasahara H., Wafula E., Depamphilis C.W., Namba S., Shirasu K. (2016). Local auxin biosynthesis mediated by a YUCCA flavin monooxygenase regulates haustorium development in the parasitic plant *Phtheirospermum japonicum*. Plant Cell.

[32] Zhang M., Ma Y.Q., Zhong W.J., Jia X.T., Wu D.R., Yu R., Ye X.X. (2015). N-P-K ratio affects exudation of germination stimulants and resistance of tobacco seedlings to broomrapes. Plant Growth Regul..

[33] Nagahashi G., Douds D.D. (2000). Partial separation of root exudate components and their effects upon the growth of germinated spores of AM fungi. Mycol. Res..

[34] Yoneyama K., Xie X.N., Kusumoto D., Sekimoto H., Sugimoto Y., Takeuchi Y., Yoneyama K. (2007). Nitrogen deficiency as well as phosphorus deficiency in sorghum promotes the production and exudation of 5-deoxystrigol, the host recognition signal for arbuscular mycorrhizal fungi and root parasites. Planta.

[35] Dobrev P.I., Hoyerova K., Petrasek J. (2017). Analytical determination of auxins and cytokinins. Auxins and Cytokinins in Plant Biology: Methods and Protocols.

[36] Weng J.K., Ye M.L., Li B., Noel J.P. (2016). Co-evolution of hormone metabolism and signaling networks expands plant adaptive plasticity. Cell.

[37] Cheng X., Flokova K., Bouwmeester H., Ruyter-Spira C. (2017). The role of endogenous strigolactones and their interaction with ABA during the infection process of the parasitic weed *Phelipanche ramosa* in tomato plants. Front. Plant Sci..

[38] Olatunji D., Geelen D., Verstraeten I. (2017). Control of endogenous auxin levels in plant root development. Int. J. Mol. Sci..

[39] Lomin S.N., Krivosheev D.M., Steklov M.Y., Arkhipov D.V., Osolodkin D.I., Schmulling T., Romanov G.A. (2015). Plant membrane assays with cytokinin receptors underpin the unique role of free cytokinin bases as biologically active ligands. J. Exp. Bot..

[40] Gajdosova S., Spichal L., Kaminek M., Hoyerova K., Novak O., Dobrev P.I., Galuszka P., Klima P., Gaudinova A., Zizkova E. (2011). Distribution, biological activities, metabolism, and the conceivable function of *cis*-zeatin-type cytokinins in plants. J. Exp. Bot..

[41] Sakakibara H. (2006). Cytokinins: Activity, biosynthesis, and translocation. Annu. Rev. Plant Biol..

[42] Raspor M., Motyka V., Ninkovic S., Dobrev P.I., Malbeck J., Cosic T., Cingel A., Savic J., Tadic V., Dragicevic I.C. (2020). Endogenous levels of cytokinins, indole-3-acetic acid and abscisic acid in *in vitro* grown potato: A contribution to potato hormonomics. Sci. Rep..

[43] Hosek P., Hoyerova K., Kiran N.S., Dobrev P.I., Zahajska L., Filepova R., Motyka V., Mueller K., Kaminek M. (2020). Distinct metabolism of *N*-glucosides of isopentenyladenine and *trans*-zeatin determines cytokinin metabolic spectrum in Arabidopsis. New Phytol..

[44] Miransari M., Abrishamchi A., Khoshbakht K., Niknam V. (2014). Plant hormones as signals in arbuscular mycorrhizal symbiosis. Crit. Rev. Biotechnol..

[45] Sugawara S., Mashiguchi K., Tanaka K., Hishiyama S., Sakai T., Hanada K., Kinoshita-Tsujimura K., Yu H., Dai X.H., Takebayashi Y. (2015). Distinct characteristics of indole-3-acetic acid and phenylacetic acid, two common auxins in plants. Plant Cell Physiol..

[46] Widhalm J.R., Dudareva N. (2015). A familiar ring to it: Biosynthesis of plant benzoic acids. Mol. Plant.

[47] Vlot A.C., Dempsey D.A., Klessig D.F. (2009). Salicylic acid, a multifaceted hormone to combat disease. Annu. Rev. Phytopathol..

[48] Halouzka R., Zeljkovic S.C., Klejdus B., Tarkowski P. (2020). Analytical methods in strigolactone research. Plant Methods.

[49] Flokova K., Shimels M., Jimenez B.A., Bardaro N., Strnad M., Novak O., Bouwmeester H.J. (2020). An improved strategy to analyse strigolactones in complex sample matrices using UHPLC-MS/MS. Plant Methods.

[50] Popova V.I.T., Stoyanova A., Nikolova V., Hristeva T., Docheva M., Nikolov N., Iliev I. (2019). Polyphenols and triterpenes in leaves and extracts from three *Nicotiana* species. J. Appl. Biol. Biotechnol..

[51] Boutet-Mercey S., Perreau F., Roux A., Clave G., Pillot J.P., Schmitz-Afonso I., Touboul D., Mouille G., Rameau C., Boyer F.D. (2018). Validated method for strigolactone quantification by ultra high-performance liquid chromatography—Electrospray ionisation tandem mass spectrometry using novel deuterium labelled standards. Phytochem. Anal..

[52] Lopez-Raez J.A., Charnikhova T., Gomez-Roldan V., Matusova R., Kohlen W., de Vos R., Verstappen F., Puech-Pages V., Becard G., Mulder P. (2008). Tomato strigolactones are derived from carotenoids and their biosynthesis is promoted by phosphate starvation. New Phytol..

[53] Louarn J., Carbonne F., Delavault P., Becard G., Rochange S. (2012). Reduced germination of *Orobanche cumana* seeds in the presence of arbuscular mycorrhizal fungi or their exudates. PLoS ONE.

[54] Lopez-Raez J.A., Charnikhova T., Fernandez I., Bouwmeester H., Pozo M.J. (2011). Arbuscular mycorrhizal symbiosis decreases strigolactone production in tomato. J. Plant Physiol..

[55] Staehelin C., Xie Z.P., Illana A., Vierheilig H. (2011). Long-distance transport of signals during symbiosis are nodule formation and mycorrhization autoregulated in a similar way?. Plant Signal. Behav..

[56] Lopez-Raez J.A., Shirasu K., Foo E. (2017). Strigolactones in plant interactions with beneficial and detrimental organisms: The Yin and Yang. Trends Plant Sci..

[57] Zagorchev L., Stoggl W., Teofanova D., Li J.M., Kranner I. (2021). Plant parasites under pressure: Effects of abiotic stress on the interactions between parasitic plants and their hosts. Int. J. Mol. Sci..

[58] Herrera-Medina M.J., Steinkellner S., Vierheilig H., Bote J.A.O., Garrido J.M.G. (2007). Abscisic acid determines arbuscule development and functionality in the tomato arbuscular mycorrhiza. New Phytol..

[59] Torres-Vera R., Garcia J.M., Pozo M.J., Lopez-Raez J.A. (2016). Expression of molecular markers associated to defense signaling pathways and strigolactone biosynthesis during the early interaction tomato-*Phelipanche ramosa*. Physiol. Mol. Plant Pathol..

[60] Torres-Vera R., Garcia J.M., Pozo M.J., Lopez-Raez J.A. (2014). Do strigolactones contribute to plant defence?. Mol. Plant Pathol..

[61] Lopez-Raez J.A., Kohlen W., Charnikhova T., Mulder P., Undas A.K., Sergeant M.J., Verstappen F., Bugg T.D.H., Thompson A.J., Ruyter-Spira C. (2010). Does abscisic acid affect strigolactone biosynthesis?. New Phytol..

[62] Etemadi M., Gutjahr C., Couzigou J.M., Zouine M., Lauressergues D., Timmers A., Audran C., Bouzayen M., Becard G., Combier J.P. (2014). Auxin perception is required for arbuscule development in arbuscular mycorrhizal symbiosis. Plant Physiol..

[63] Shaul-Keinan O., Gadkar V., Ginzberg I., Grunzweig J.M., Chet I., Elad Y., Wininger S., Belausov E., Eshed Y., Arzmon N. (2002). Hormone concentrations in tobacco roots change during arbuscular mycorrhizal colonization with *Glomus intraradices*. New Phytol..

[64] Kieber J.J., Schaller G.E. (2018). Cytokinin signaling in plant development. Development.

[65] Goyet V., Billard E., Pouvreau J.B., Lechat M.M., Pelletier S., Bahut M., Monteau F., Spichal L., Delavault P., Montiel G. (2017). Haustorium initiation in the obligate parasitic plant *Phelipanche ramosa* involves a host-exudated cytokinin signal. J. Exp. Bot..

[66] Kirilova I., Hristeva T., Denev I. (2019). Identification of seeds of *Phelipanche ramosa*, *Phelipanche mutelii* and *Orobanche cumana* in the soils from different agricultural regions in Bulgaria by molecular markers. Biotechnol. Biotechnol. Equip..

[67] Prerostova S., Dobrev P.I., Knirsch V., Jarosova J., Gaudinova A., Zupkova B., Prasil I.T., Janda T., Brzobohaty B., Skalak J. (2021). Light quality and intensity modulate cold acclimation in Arabidopsis. Int. J. Mol. Sci..

[68] Dobrev P.I., Kaminek M. (2002). Fast and efficient separation of cytokinins from auxin and abscisic acid and their purification using mixed-mode solid-phase extraction. J. Chromatogr. A.

[69] Dobrev P.I., Vankova R. (2012). Quantification of abscisic acid, cytokinin, and auxin content in salt-stressed plant tissues. Methods Mol. Biol..

[70] Koske R.E., Gemma J.N. (1989). A modified procedure for staining roots to detect VA-mycorrhizas. Mycol. Res..

[71] Giovannetti M., Mosse B. (1980). An evaluation of techniques for measuring vesicular arbuscular mycorrhizal infection in roots. New Phytol..

