# Supplementary material for: Hormonomic Changes Driving the Negative Impact of Broomrape on Plant Host Interactions with Arbuscular Mycorrhizal Fungi
Source: Int J Mol Sci. 2021 Dec 20;22(24):13677. doi: 10.3390/ijms222413677 (PMC8708155; doi:10.3390/ijms222413677)
Supplement: Supplementary file 1 [file ijms-22-13677-s001.zip › Mishev et al_Suppl Mat_IJMS.pdf]

Supplementary Materials

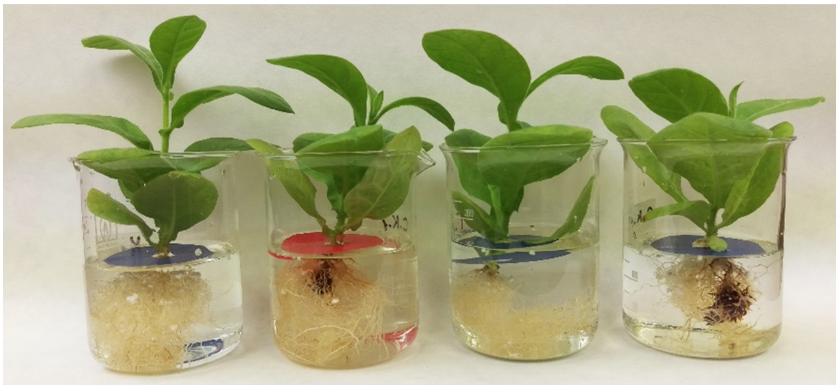

Tobacco (control)	Tobacco + broomrape	Tobacco + AMF	Tobacco + AMF + broomrape

Figure S1. Experimental setup for collection of tobacco root exudates. After perlite removal, intact plants were placed in glass beakers and the root system was submerged in distilled water. Plastic discs were used to prevent any contact of the leaves with the water surface. The water solution was collected following two days of incubation under controlled conditions (see Subsection 4.2).

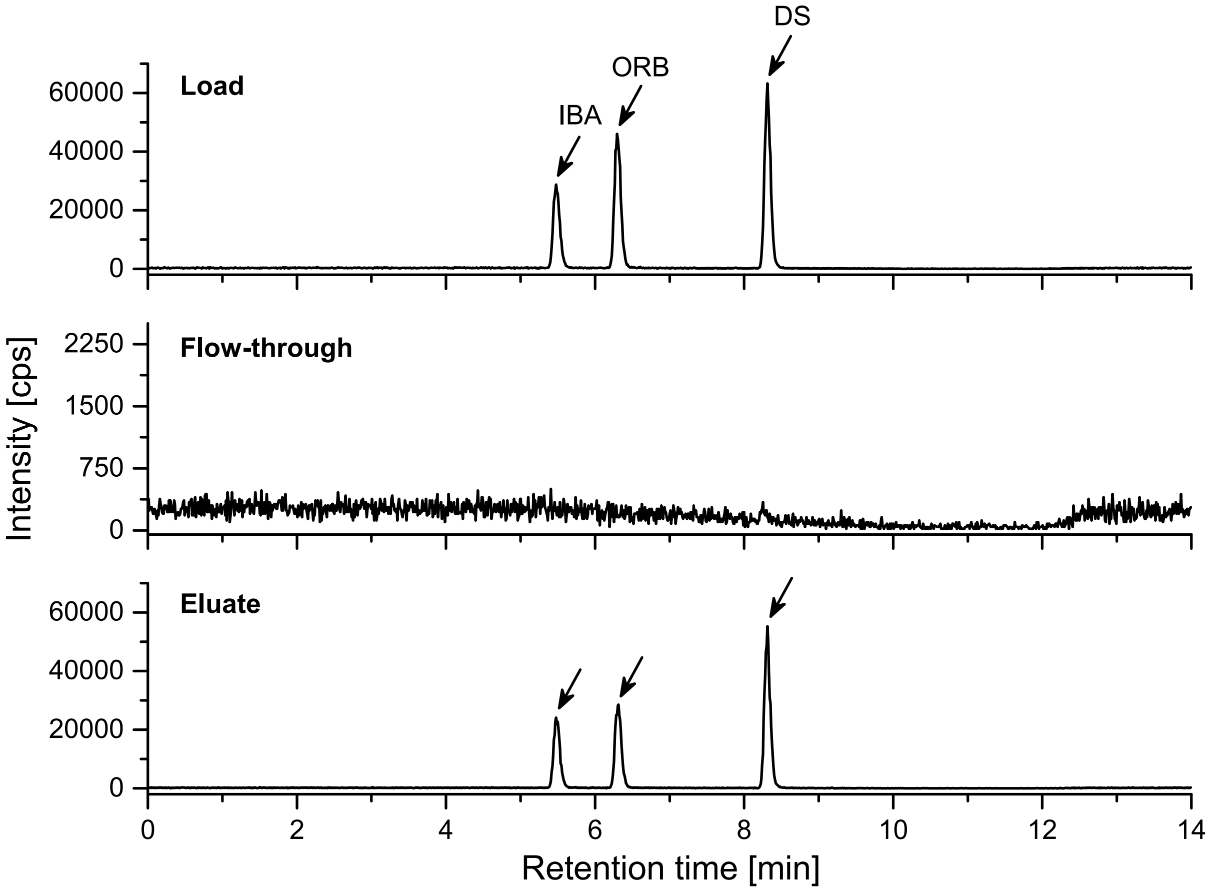

Figure S2. Capacity of reversed phase Sep-Pak® Plus columns with silica-based C18 sorbent to retain strigolactones (SLs). The total ion chromatogram shows the presence of the SL standards 2′-epi-orobanchol (ORB) and 5-deoxystrigol (DS), as well as the auxin standard indole-3-butyric acid (IBA) in the water solution prior to loading onto the column as well as in the final acetone eluate, but not in the flow-through fraction.

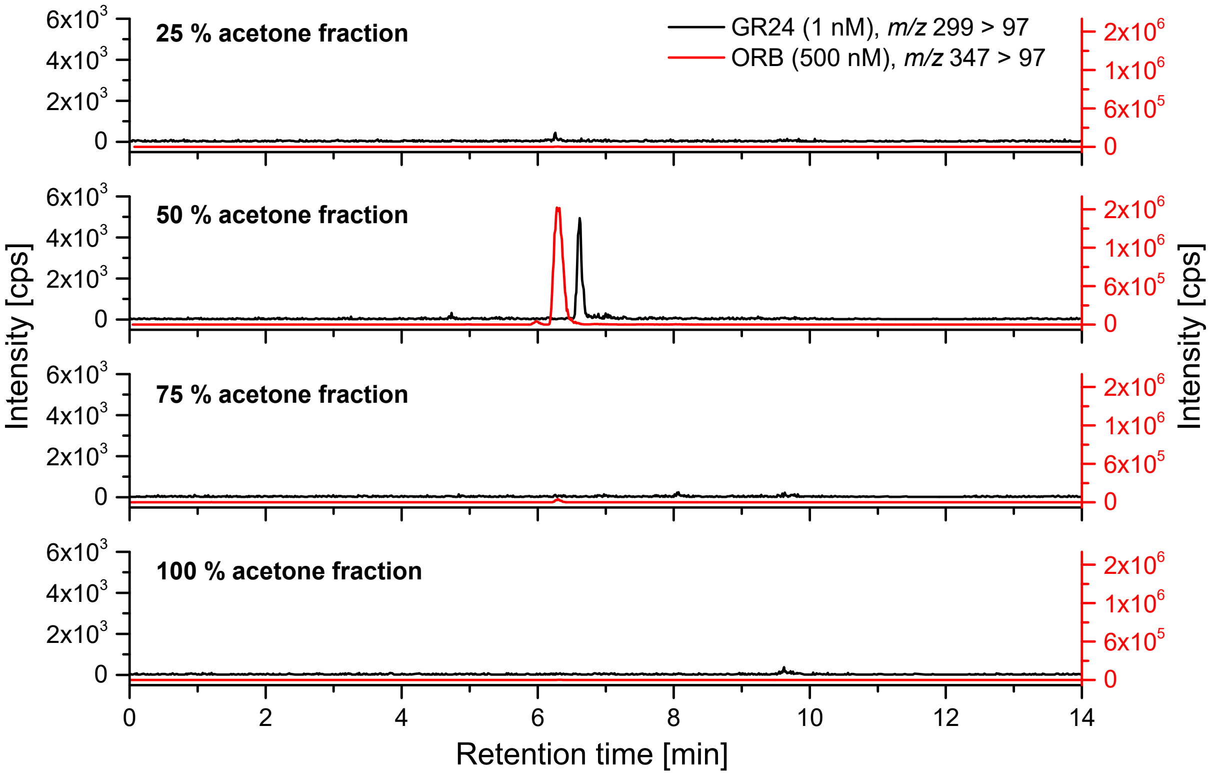

Figure S3. Strigolactone recovery after stepwise elution with increasing concentrations of acetone. Water solution of GR24 (1 nM) and 2′-epi-orobanchol (ORB, 500 nM) was passed through preconditioned Sep-Pak® Plus columns, followed by collection of the compound fractions eluted from the columns consecutively with 25 %, 50 %, 75 %, and 100 % acetone. The multiple reaction monitoring (MRM) chromatograms show the transitions of *m*/*z* 299 > 97 for GR24 (black line) and *m*/*z* 347 > 97 for ORB (red line).
